# 非小细胞肺癌原发灶与相应转移灶之间*EGFR*基因突变状况的不一致性研究

**DOI:** 10.3779/j.issn.1009-3419.2011.06.07

**Published:** 2011-06-20

**Authors:** 勤 方, 亮 张, 思愚 王, 伟 区

**Affiliations:** 510060 广州，中山大学附属肿瘤医院胸科 Department of Toracic Surgery, Cancer Center of Sun Yat-sen University, Guangzhou 510060, China

**Keywords:** 肺肿瘤, 原发灶, 转移灶, *EGFR*基因, Lung neoplasms, Primary tumor, Corresponding metastases, Epidermal growth factor receptor

## Abstract

**背景与目的:**

表皮生长因子受体（epidermal growth factor receptor, EGFR）突变是晚期非小细胞肺癌（non-small cell lung cancer, NSCLC）患者获益于酪氨酸激酶抑制剂（tyrosine kinase inhibitor, TKI）治疗的预测因子，本研究旨在探讨NSCLC原发灶与相应转移灶之间*EGFR*基因突变状况的不一致性。

**方法:**

应用TaqMan RT-PCR的方法检测35例病理确诊为NSCLC患者原发灶和相应转移灶的*EGFR*基因突变状况。

**结果:**

原发肺癌病灶中有29例为*EGFR*基因突变型，余下6例为EGFR野生型。35例转移灶中18例为*EGFR*基因突变型，17例为EGFR野生型。35对配对标本中，11对（31.43%）标本出现原发灶*EGFR*基因突变，而转移灶为*EGFR*基因野生型，18对原发灶及转移灶均为*EGFR*基因突变型，且突变具体位点相同，6对原发灶及转移灶均为*EGFR*基因野生型。NSCLC原发灶与转移灶的*EGFR*基因表达不一致率为31.43%（11/35, *P*=0.008）。

**结论:**

NSCLC原发灶与转移灶的*EGFR*基因表达存在不一致性。

## Abstract (en)

**Background and objective:**

Epidermal growth factor receptor (*EGFR*) gene mutation assay has been applied to select the chemosensitive patients for tyrosine kinase inhibitor (TKI) treatment widely. The aim of this study is to determine the discordance of *EGFR* mutations between primary and corresponding metastatic tumors in non-small cell lung cancer (NSCLC).

**Methods:**

Thirty five paired primary tumors and corresponding metastases from Cancer Center of Sun Yatsen University were evaluated for the *EGFR* mutations by TaqMan RT-PCR analysis.

**Results:**

*EGFR* mutations were detected in 29 of 35 primary tumors and in 18 of 35 corresponding metastases. 31.43% (11 of 35, *P*=0.008) showed discordance in *EGFR* mutations between primary tumors and corresponding metastases. The EGFR mutation status was consistent in 68.57% (24 of 35) patients.

**Conclusion:**

*EGFR* mutations were discordant between the primary tumor and the corresponding metastases in NSCLC.

肺癌是全世界癌症死亡的常见病因之一，非小细胞肺癌（non-small cell lung cancer, NSCLC）占原发性肺癌的80%，而其中55%的患者在诊断时就已经是局部晚期或远处转移^[1]^。虽然以铂类为基础的联合化疗已经提高了中位生存时间，但这部分患者的预后仍然很差，平均5年生存率不足5%^[2]^。近年来酪氨酸激酶抑制剂（tyrosine kinase inhibitor, TKI）分子靶向治疗问世，使晚期NSCLC治疗取得较大的突破，具有疗效明显、毒副作用小、耐受性好的优势，逐渐成为研究热点之一^[3-5]^。

在探索哪些患者能获益于TKI治疗的过程中，研究者发现表皮生长因子受体（epidermal growth factor receptor, *EGFR*）基因突变是晚期NSCLC患者获益于TKI治疗的预测因子^[6-10]^。*EGFR*基因定位于人第7号染色体短臂，由118 kb组成，包括28个外显子，其中第18-21号外显子编码TK区域。*EGFR*基因的众多突变中，89%都位于第19、21号外显子；这两个位点突变已经被证实与TKI治疗有效有关^[3]^。数项前瞻性及回顾性研究^[11-16]^证实*EGFR*基因突变阳性的患者对TKI（吉非替尼或厄洛替尼）治疗有效率为70%-80%，能有效延长无进展生存时间（progression-free survival, PFS）、提高生活质量，其中位生存时间达20个月-30个月。

在局部或者晚期的NSCLC患者中，决定是否使用TKI治疗时检测*EGFR*基因突变的标本多是选取原发灶或者转移灶。在临床工作中，由于穿刺活检或经支气管镜活检获取原发灶的组织量不够，采用转移灶特别是体表淋巴结作为EGFR突变状况检测较多。然而目前在原发灶与转移灶之间*EGFR*基因突变状况是否存在一致性尚不明确。Park^[17]^研究发现在原发肿瘤与转移淋巴结之间，用直接测序法检测*EGFR*基因突变状况的不一致率为11.9%（12/101），而同时用异源双链分析法检测其不一致率为16.8%（17/101）。最近同类研究也显示在NSCLC原发灶与相应转移灶之间*EGFR*基因突变状况的不一致率分别为27%（18/67）^[18]^、33%（16/45）^[19]^、28%（7/25）^[20]^。

本研究旨在通过比较NSCLC原发灶与转移灶之间*EGFR*基因突变状况，探索二者之间是否存在不一致性。

## 1 材料与方法

### 1.1 临床资料

研究的入组标准如下：①完整切除原发病灶的NSCLC患者；②相应的转移病灶有足够多的癌组织可行*EGFR*基因突变检测；③既往未接受过TKI治疗；④患者年龄>18岁。2008年1月-2010年3月共收集了40对NSCLC原发灶及相应转移灶标本，其中5对标本因转移灶标本量太少，无法行*EGFR*基因突变检测而排除，最后共35对标本入组。

记录病例的临床病理资料，包括性别、年龄、吸烟史、肿瘤大小、病理分型、肿瘤分化情况、术后的病理分期等。术后的病理分期根据IASLC 2009年制定的NSCLC分期标准进行评定^[21]^，肿瘤的分期与肿瘤发生时的诊断一致。肿瘤组织的病理分型根据2005年WHO标准进行评定^[22]^。吸烟史在患者第一次就诊时获得，吸烟状态分为：①不吸烟者（一生中吸烟 < 100支）；②曾经吸烟者（术前12个月内不再吸烟）；③现时吸烟者（术前12个月内有吸烟并且≥100支）^[9]^。所有患者已经被告知并同意使用其标本用于分子和病理学分析。该试验已获得医院伦理委员会的批准。

### 1.2 实验方法

本研究中所使用的*EGFR*基因检测方法为TaqMan RT-PCR ^[23]^。本次应用的EGF R基因检测试剂盒共检测EG F R基因1 9号外显子三种碱基缺失突变（A: Nucleotide 2240-2251del, Amino acid L747-A750del; B: Nucleotide 2235-2249del, Amino acid E746-A750del; C: Nucleotide 2240-2257del, Amino acid L747-S752del）及21号外显子2种碱基置换突变（D: Nucleotide 2573 T>G, Amino acid L858R; E: Nucleotide 2582 T>A, Amino acid L861Q）共5个最常见突变位点。

实验大致过程如下：①两位病理科医生分别在显微镜下核对石蜡组织的H E染色玻片，选取肿瘤组织>80%的石蜡块；②切取每片10 μm厚的蜡片4张；③使用TIANamp基因组DNA提取试剂盒（TIANGEN BIOTECH, 北京)从切片中提取出原发肺癌组织和转移灶的基因组DNA；④进行DNA提取物的验证：甘油醛-3-磷酸脱氢酶（glyceraldehyde-3-phosphate dehydrogenase, GAPDH）管家基因（300 bp）PCR扩增，*GAPDH*基因扩增产物在琼脂糖凝胶电泳后紫外灯照射下进行拍照（没有PCR扩增条带的标本需再次切片，再次提取DNA）；⑤把成功提取的DNA放入EP管中并分别加入*EGFR*基因检测试剂盒（GP Medical Technologies, Beijing, China）中的正向引物、反向引物、探针等；⑥把标本放入ABI7900 RT-PCR机（Esco Technologies, Inc., St. Louis, MO, USA）上进行扩增^[24]^；⑦应用SDS 2.0软件对扩增曲线进行分析并判断EGFR基因突变阴、阳性。

### 1.3 统计方法

统计分析采用SPSS 16.0统计学软件。计数资料分析采用*Fisher’s*检验。*P* < 0.05为有统计学差异。

## 2 结果

### 2.1 患者的一般情况

35对NSCLC病例的配对标本中，原发灶均是已经完整切除的肺部病灶，35例配对的转移灶中，29例是纵隔淋巴结转移灶（9枚隆突下淋巴结，8枚主动脉下淋巴结，8枚下气管旁淋巴结，4枚上气管旁淋巴结），4例是脑转移病灶，2例是锁骨上淋巴结转移灶。所有病例在获得肿瘤标本前未曾接受过EGFR靶向治疗。病例的一般情况数据如下：中位年龄为57岁（38岁-72岁）；女性14例（40%），男性21例（60%）；病理学类型为：鳞癌1例（2.9%），腺鳞癌3例（8.6%），腺癌31例（88. 5%）；肿瘤分期：Ⅲa期24例（68.6%），Ⅲb期3例（8.6%），Ⅳ期8例（22.8%）；吸烟史：不吸烟者16例（45.8%），既往吸烟者13例（37.1%），现时吸烟者6例（17.1%）。

### 2.2 NSCLC原发病灶和转移灶的*EGFR*基因突变状况

*EGFR*基因突变RT-PCR扩增曲线见图 1。在这项研究中，原发肺癌病灶中有29例（29/35, 82.86%）为*EGFR*基因突变型，余下6例为EGFR野生型。29例原发肺癌病灶*EGFR*突变类型分别为第19号外显子突变15例、第21号外显子突变14例。其中第19号外显子突变中均为E746-A750缺失；第21号外显子突变中13例为第858位密码子碱基替换突变，1例为第861位密码子碱基替换突变。

**1:**
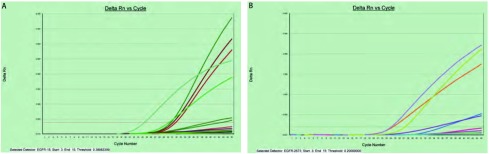
*EGFR*基因突变检测RT-PCR扩增曲线。A：19号外显子E746-A750del；B：21号外显子L858R E。 RT-PCR amplification curve of *EGFR* gene mutations analysis. A: exon 19. Amino acid L747-A750del; B: exon 21, Nucleotide 2573 T>G.

35例转移灶中18例（18/35, 51.43%）为*EGFR*基因突变型，17例为EGFR野生型。18例*EGFR*基因突变型转移灶的具体突变类型为：第19号外显子突变11例、第21号外显子突变7例。其中第19号外显子突变中均为E746-A750缺失；第21号外显子突变中均为第858位密码子碱基替换突变（表 1）。

**1:** 原发灶与相应转移灶的*EGFR*基因突变状况 *EGFR* mutations in primary and corresponding metastatic tumors

Case	Histology	Primary lung tumor		Metastatic sites	Metastatic tumor	
		Types of mutations	Mutation site		Types of mutations	Mutation site
1	ADC	exon 21	2582 T>A	Subaortic LN	wild	wild
2	ADC	exon 21	2573 T>G	Brain	21 exon	2573 T>G
3	ASC	exon 21	2573 T>G	Subaortic LN	wild	wild
4	ADC	exon 21	2573 T>G	Lower paratracheal LN	wild	wild
5	ADC	exon 21	2573 T>G	Upper paratracheal LN	wild	wild
6	ADC	exon 21	2573 T>G	Lower paratracheal LN	21 exon	2573 T>G
7	ADC	exon 21	2573 T>G	Subcarinal LN	wild	wild
8	ADC	exon 21	2573 T>G	Lower paratracheal LN	21 exon	2573 T>G
9	ADC	exon 21	2573 T>G	Subaortic LN	21 exon	2573 T>G
10	ADC	exon 21	2573 T>G	Subaortic LN	21 exon	2573 T>G
11	ADC	exon 21	2573 T>G	Subcarinal LN	21 exon	2573 T>G
12	ADC	exon 21	2573 T>G	Subcarinal LN	21 exon	2573 T>G
13	ADC	exon 21	2573 T>G	Lower paratracheal LN	wild	wild
14	ADC	exon 21	2573 T>G	Lower paratracheal LN	wild	wild
15	ASC	exon 19	E746-A750	Lower paratracheal LN	19 exon	E746-A750
16	ADC	exon 19	E746-A750	Subcarinal LN	19 exon	E746-A750
17	ADC	exon 19	E746-A750	Upper paratracheal LN	19 exon	E746-A750
18	ASC	exon 19	E746-A750	Subaortic LN	19 exon	E746-A750
19	ADC	exon 19	E746-A750	Subaortic LN	19 exon	E746-A750
20	ADC	exon 19	E746-A750	Subcarinal LN	19 exon	E746-A750
21	ADC	exon 19	E746-A750	Subcarinal LN	wild	wild
22	ADC	exon 19	E746-A750	Lower paratracheal LN	19 exon	E746-A750
23	ADC	exon 19	E746-A750	Subaortic LN	19 exon	E746-A750
24	ADC	exon 19	E746-A750	Subcarinal LN	wild	wild
25	ADC	exon 19	E746-A750	Lower paratracheal LN	19 exon	E746-A750
26	SQC	exon 19	E746-A750	Subaortic LN	wild	wild
27	ADC	exon 19	E746-A750	Subcarinal LN	19 exon	E746-A750
28	ADC	exon 19	E746-A750	Subcarinal LN	wild	wild
29	ADC	exon 19	E746-A750	Upper paratracheal LN	19 exon	E746-A750
30	ADC	wild	wild	Supraclavicular LN	wild	wild
31	ADC	wild	wild	Supraclavicular LN	wild	wild
32	ADC	wild	wild	Brain metastases	wild	wild
33	ADC	wild	wild	Brain metastases	wild	wild
34	ADC	wild	wild	Upper paratracheal LN	wild	wild
35	ADC	wild	wild	Brain	wild	wild
ADC: adenocarcinoma; SQC: squamous cell carcinoma; ASC: adenosquamous carcinoma; LN: lymph node.						

### 2.3 原发NSCLC病灶和相应转移灶的*EGFR*基因突变状况比较

6例NSCLC原发灶为*EGFR*基因野生型的病例，其相应的转移灶分别为：3例脑转移病灶、2例锁骨上淋巴结转移灶和1例上气管旁淋巴结转移灶，这6例转移灶的*EGFR*基因均为野生型，与原发病灶一致。

29例原发灶*EGFR*基因突变型的病例中，只有18例*EGFR*基因突变，而其余11例转移灶为*EGFR*基因野生型。18例EGFR突变型的转移灶中，11例为第19号外显子突变，病灶分别为：3例下气管旁淋巴结转移灶、3例隆突下淋巴结转移灶、3例主动脉下淋巴结转移灶及2例上气管旁淋巴结转移灶，第19号外显子突变均为E746-A750缺失；另外7例为第21号外显子突变，其相应转移灶分别为：2例下气管旁淋巴结转移灶、2例隆突下淋巴结转移灶、2例主动脉下淋巴结转移灶及1例脑转移灶，第21号外显子突变均为第858位密码子碱基替换突变。转移灶的EGFR基因突变位点与原发病灶的突变位点一致。

11例（11/35, 31.43%, *P*=0.008）原发灶*EGFR*基因突变阳性而转移灶为*EGFR*基因野生型的患者其转移灶分别为：4例隆突下淋巴结转移灶、3例下气管旁淋巴结转移灶、3例主动脉下淋巴结转移灶。其原发灶*EGFR*基因突变类型为：第19号外显子突变4例（均为E746-A750缺失）、第21号外显子突变7例（6例为第858位密码子碱基替换突变，1例为第861位密码子碱基替换突变），其相应转移灶均为*EGFR*基因野生型，与原发灶*EGFR*基因表达不一致。

35例原发NSCLC病灶与相应转移灶配对标本中，11例（31.43%, *P*=0.008）标本出现原发灶*EGFR*基因突变，而转移灶为*EGFR*基因野生型，18对原发灶及转移灶均为*EGFR*基因突变型，且突变具体位点相同，6对原发灶及转移灶均为*EGFR*基因野生型。研究结果显示转移灶与原发灶的*EGFR*基因表达存在不一致性，其不一致率为31.43%（11/35, *P*=0.008）。

## 3 讨论

在晚期NSCLC患者中*EGFR*突变者对TKI治疗的反应率及PFS均较EGFR野生型者明显升高。检测*EGFR*基因突变来筛选获益于TKI治疗的患者已经推广使用。目前转移病灶的*EGFR*基因状况是否与原发病灶保持一致尚不明确。当晚期NSCLC患者在决定是否使用EGFR TKI治疗时，进行*EGFR*基因突变状况检测，是否原发病灶与转移病灶同时检测更合适，也无法定论。

本研究比较了NSCLC原发病灶与相应转移病灶之间*EGFR*基因突变状况的一致性，共检测了35对配对的原发肺癌病灶与相应转移灶的*EGFR*基因。研究结果显示转移灶与原发灶的*EGFR*基因表达存在不一致性。入组病例中有11例（31.43%）出现原发灶*EGFR*基因突变，而其对应的转移灶为*EGFR*基因野生型。这一发现与最近几项同类研究的结果相似，Park和Gow等研究结果显示在NSCLC原发灶与相应转移灶之间*EGFR*基因表达的不一致率分别为11.9%（12/101，直接测序法），16.8%（17/101，异源双链分析法）^[17]^、27%（18/67，TaqMan RT-PCR法) ^[18]^、33%（16/45，免疫组化）^[19]^、28%（7/25，直接测序法）^[20]^。各个实验检测*EGFR*基因突变的方法不同及病例入组标准的差异可能是造成不一致率存在差异的原因。

然而，某些研究却发现相反的结果。Matsumoto在研究中有8例肺腺癌脑转移灶患者可找到相应的肺部原发病灶，其中6例脑转移灶和肺部原发病灶均为*EGFR*基因突变型，表达一致^[25]^。另一个研究^[26]^对比8例肺癌患者原发灶与转移灶的基因表达，*EGFR*基因、*KRAS*、*P53*在原发灶与转移灶之间都保持了一致性。然而这些研究的入组病例数都很少。

EGFR TKI治疗在*EGFR*基因突变型晚期NSCLC患者中有明显疗效，能有效延长PFS、提高生活质量^[11-16]^。TKI治疗是否是晚期肺癌患者合适的治疗，根据*EGFR*突变类型来选择是关键，然而*EGFR*基因在原发灶与转移灶之间存在着不一致性，所以如果对肺癌原发灶及转移灶同时进行*EGFR*基因检测，可能对患者的治疗有更好的指导意义。

总之，本实验发现在NSCLC原发病灶与转移灶之间的EGFR基因状况的不一致率为31.43%。这种原发灶与转移灶EGFR基因状况的不一致性可能对晚期NSCLC患者选择EGFR TKI治疗有一定程度的影响，同时也是将来相关临床试验设计时需考虑到的因素之一，尚有待大样本量的研究来进一步验证其临床意义。
